# Supercritical Fluids: A Promising Technique for Biomass Pretreatment and Fractionation

**DOI:** 10.3389/fbioe.2020.00252

**Published:** 2020-04-23

**Authors:** Estephanie Laura Nottar Escobar, Thiago Alessandre da Silva, Cleverton Luiz Pirich, Marcos Lúcio Corazza, Luiz Pereira Ramos

**Affiliations:** ^1^Applied Kinetics and Thermodynamics Laboratory, Department of Chemical Engineering, Federal University of Paraná, Curitiba, Brazil; ^2^Department of Chemistry, Research Center in Applied Chemistry, Federal University of Paraná, Curitiba, Brazil

**Keywords:** lignocellulosic biomass, biomass pretreatment and fractionation, supercritical fluids, biorefinery, biofuels

## Abstract

Lignocellulosic biomasses are primarily composed of cellulose, hemicelluloses and lignin and these biopolymers are bonded together in a heterogeneous matrix that is highly recalcitrant to chemical or biological conversion processes. Thus, an efficient pretreatment technique must be selected and applied to this type of biomass in order to facilitate its utilization in biorefineries. Classical pretreatment methods tend to operate under severe conditions, leading to sugar losses by dehydration and to the release of inhibitory compounds such as furfural (2-furaldehyde), 5-hydroxy-2-methylfurfural (5-HMF), and organic acids. By contrast, supercritical fluids can pretreat lignocellulosic materials under relatively mild pretreatment conditions, resulting in high sugar yields, low production of fermentation inhibitors and high susceptibilities to enzymatic hydrolysis while reducing the consumption of chemicals, including solvents, reagents, and catalysts. This work presents a review of biomass pretreatment technologies, aiming to deliver a state-of-art compilation of methods and results with emphasis on supercritical processes.

## Introduction

Ethanol is one of the main biomass-originated fuels that can be produced from food crops rich in carbohydrates such as sucrose and starch. However, the production of ethanol from edible feedstocks has raised ethical questions that are related to food security, including the availability of arable land for farming and a marked dispute causing an inevitable rise in food prices. Thus, cellulosic ethanol emerges as a sustainable alternative for the world energy matrix because its production is based on non-edible harvesting or processing residues derived from forestry and agro-industrial activities, along with energy crops that can be cultivated in marginal lands (Mohr and Raman, 2013).

Lignocellulosic materials are composed of cellulose, hemicelluloses, and lignin and this complex matrix have been designed by nature to resist the impact of weather and/or biological degradation. In the context of biorefineries, this natural recalcitrance affects the accessibility of plant polysaccharides to chemical and enzymatic treatments that promote hydrolysis of cellulose and hemicelluloses to produce fermentable sugars (Himmel et al., 2007).

Chemical hydrolysis is based on acid catalysts and it is faster than enzymatic hydrolysis. However, in the chemical pathway, one needs to neutralize process streams and the formulated medium before fermentation. Besides that, hydrolysis and fermentation inhibitors are produced depending on the reaction conditions. These are mostly originated from carbohydrate dehydration and condensation reactions involving lignin. Problems with equipment corrosion and effluent disposal are also a drawback, requiring specific materials for reactor design and complex waste treatment installations. By contrast, enzymatic hydrolysis presents high specificity, low energy requirements and no release of inhibitory compounds, but the reaction rates are slow due to biomass recalcitrance. Therefore, lignocellulosic materials must be submitted to a suitable pretreatment technique prior to enzymatic hydrolysis to reduce the enzyme requirements for optimal performance and to increase their inherently low reaction rates (Zheng et al., 2009; Chen et al., 2017; Kumar and Sharma, 2017).

Several pretreatment techniques have been studied to improve the conversion of lignocellulosic materials into sustainable fuels, value-added chemicals and functional materials (Rajan and Carrier, 2014; Zhang et al., 2016; Qi et al., 2017). Pretreatment normally enhances substrate accessibility by breaking down the hemicellulose and/or lignin interface and expose the cellulose matrix to the concerted action of cellulolytic enzymes (Mood et al., 2013).

Traditional pretreatment methods involve physical processes to increase surface area and pore volume, chemical processes to remove hemicelluloses and/or lignin, and biological processes to degrade plant macromolecular components by the action of enzymes or microorganisms (Agbor et al., 2011). The combination of two or more of these techniques generate efficient processes that result in high sugar yields and enzymatic hydrolysis rates. Pressurized fluids have also been proposed as a promising pretreatment method. In this field, the emphasis has been on supercritical CO_2_ (scCO_2_) because it can be applied as a fast pretreatment technique that dispenses subsequent separation processes because this compressed solvent can be easily recovered from the medium only by expansion to ambient conditions (Zheng et al., 1998; Narayanaswamy et al., 2011; Melo et al., 2014; Attard et al., 2016).

Many reviews on lignocellulosic biomass utilization have been published in the literature, most of them providing a breakdown of traditional biomass pretreatment techniques and their effects over biomass accessibility to chemical and biological conversion processes, or reporting the main effects of pretreatment on the biomass structure and chemical composition (Agbor et al., 2011; Mood et al., 2013; Silveira et al., 2015a; Ponnusamy et al., 2019). Some of these publications reviewed supercritical processes for biomass applications. Rostagno et al. (2015) reported a review on subcritical and supercritical technologies applied to second generation ethanol. Additionally, Morais et al. (2015) reported a broad review on the use of scCO_2_ and CO_2_ assisted hydrothermal strategies for biomass processing, as well as the utilization of the aforementioned technologies in the production of biochemicals through the conversion of proteins and polysaccharides. Soh and Eckelman (2016) evaluated key metrics for green solvents applicable to biomass processing. A review on the extraction of biofuels such as bio-oils from lignocellulosic and algal biomass was also reported, mostly employing supercritical ethanol, methanol or acetone (Akalin et al., 2017). Lignin fractionation and depolymerization under different strategies, including the use of supercritical fluids, was a theme reviewed by Gillet et al. (2017). Also, a review on both catalytic and non-catalytic lignocellulose deconstruction in ethanol, together with a critical revision on the application of analytical techniques (GC-MS, 1D, and 2D NMR spectroscopy, and elemental analysis), was published by Tekin et al. (2018). Liu et al. (2019) reported an extensive review on biomass utilization with green technologies, including microwave and ultrasonic irradiation, ionic liquids, deep eutectic solvents, electric field processes, and supercritical fluids, among other approaches. Nevertheless, no critical review has been made available on the utilization of sundry supercritical fluids in the light of the biorefinery concept. On the basis of this, the objective of this work was to focus on the discussion of the impact of different supercritical fluids and processes on the conversion of lignocellulosic materials to fuels, platform chemicals and materials in the absence and presence of modifiers such as green solvents and acid catalysts.

## Lignocellulosic Matrix Structure

Woody and non-woody agro-industrial residues are mainly composed of macromolecular components such as cellulose, hemicelluloses, and lignin. Inorganic materials (ashes) and solvent extractable components are also present in variable amounts depending on the biomass type, cultivation, harvesting, handling and processing conditions. These extractable materials may contain terpenes, resins, phenols, oils, fats, waxes, pectins, and proteins (McMillan, 1994).

### Cellulose

**FIGURE 1:**
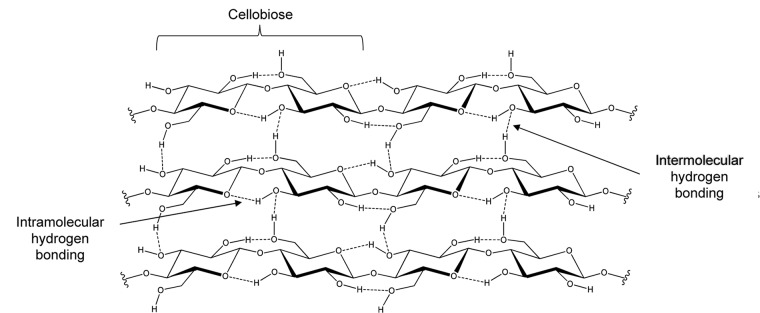
Cellobiose structure and the intricated network of intra- and intermolecular hydrogen bonding that holds the crystalline structure of cellulose together.

Cellulose is a homopolysaccharide that is insoluble in water and most organic solvents (Medronho et al., 2012), being the most abundant biopolymer on Earth and an excellent raw material for the production of fuels and chemicals by hydrolysis and fermentation. This homopolymer is composed of D-glucopyranosyl residues linked to one another by β-1,4-glycosidic bonds as shown in Figure 1. The structural analysis of cellulose chains indicated that the disaccharide cellobiose (4-*O*-β-D-glucopyranosyl-β-D-glucopyranose) is the repeating conformational unit, while glucose is its repeating building block (Ramos, 2003).

Cellulose chains are assembled in amorphous and crystalline fiber aggregates. The latter has a high level of molecular organization whose structure is maintained by a network of intra- and intermolecular hydrogen bonding that confers great resistance to enzymatic hydrolysis. The degree of polymerization (DP), which represents the number of glucosyl residues found in the linear chain, is an important structural factor to evaluate the mode of action of cellulolytic enzymes; however, crystallinity appears to have a pronounced effect on cellulose accessibility (Puri, 1984; Zheng et al., 2009).

Understanding the mode of action of cellulases on chemically defined cellulosic substrates is from the mathematical viewpoint a critical parameter for scale-up operations. However, enzymatic hydrolysis of cellulose requires a series of complex events to be effective. First, the enzymes need to adsorb onto the cellulose surface and fit a cellulose chain into the active site. This mode of action can be influenced by several factors such as substrate total solids, enzyme loading (including activity ratio and optimal synergism), buffering and stirring conditions, all of them affecting the enzyme kinetics (Wang and Feng, 2010). On the other hand, differences in the ratio of amorphous and crystalline regions in the cellulose bulk contribute to a specific reaction rate behavior. The most acceptable kinetic model is the fractal, in which the reaction rate (k) is dependent on time (Wojtusik et al., 2016, 2018; Fockink et al., 2017). The model can be simply described as a sum of different pseudo-first order reactions that are due to the distribution of enzymes onto the cellulose surface (Väljamäe et al., 2003).

Another successful kinetic model developed to describe the enzymatic hydrolysis of cellulose is the Holtzapple–Caram–Humphrey-1 mechanistic model (HCH-1) (Holtzapple et al., 1984). Basically, the HCH-1 model considers an adsorption of free enzyme onto a free cellulose surface forming an enzyme-cellulose complex. This complex promotes the hydrolysis of cellulose to obtain soluble products. Until the formation of the complex, it is assumed that enzymes could be in an inhibited state and the reaction velocity is proportional to the concentration of uninhibited enzyme-substrate complex. Nonetheless, this original model is only applied to short-term enzymatic hydrolysis that does not consider factors that decrease velocity rates, such as enzyme deactivation by lignin, end-product inhibition and changes in substrate reactivity, accessibility and synergism between enzymes (Bansal et al., 2009). Recently, modifications of HCH-1 model were developed in order to follow long-term enzymatic hydrolysis of cellulose up to 10-day experiment without considering lignin inhibition (Liang et al., 2019). Hence, this assumption was probably supported by the blocking of lignin inhibition by β-glucosidases that are found in excess in the most recent commercial cellulase preparations.

### Hemicelluloses

**FIGURE 2:**
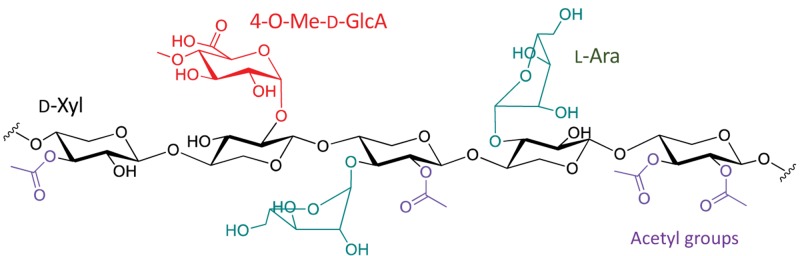
Grass xylans as an example of hemicellulose structure.

Hemicelluloses are composed of a polysaccharide matrix (Figure 2) linked to cellulose by hydrogen bonds and to lignin by covalent bonds. These polysaccharides are made up of branched chains that may contain pentoses (xylose, arabinose), hexoses (mannose, glucose, galactose), uronic acids (glucuronic and 4-*O*-methyl-glucuronic acids) and acetyl groups, having a molecular mass smaller than that of cellulose and side chains that are easily hydrolyzable due to their higher chemical accessibility (Saha, 2003). Xylans are essential polysaccharide for the cell wall structure, representing one of the most abundant components of hemicelluloses. Found mainly in hardwoods and grasses, they are constituted of heteropolysaccharides with a homopolymeric linear main chain formed by β-D-xylopyranosyl units that are partially acetylated and decorated with arabinosyl and glucuronosyl substituents. Conifers, in turn, mainly contain glucomannans that are partially water-soluble and composed of mannosyl and glucosyl in the main chain, while acetyl and galactosyl groups are found in their side chains (Saha, 2003; Tester and Al-Ghazzewi, 2017).

### Lignin

Lignin is an ether-linked biopolymer composed of three main monolignols that arise from the following cinnamic acids, *p*-coumarylic, coniferylic, and sinapylic, which are responsible for the formation of its *p*-hydroxyphenyl, guaiacyl, and syringyl units (Ponnusamy et al., 2019). Lignin confers rigidity and impermeability to cell walls and together with hemicelluloses makes up the non-cellulosic portion of lignocellulose (Agbor et al., 2011).

**FIGURE 3:**
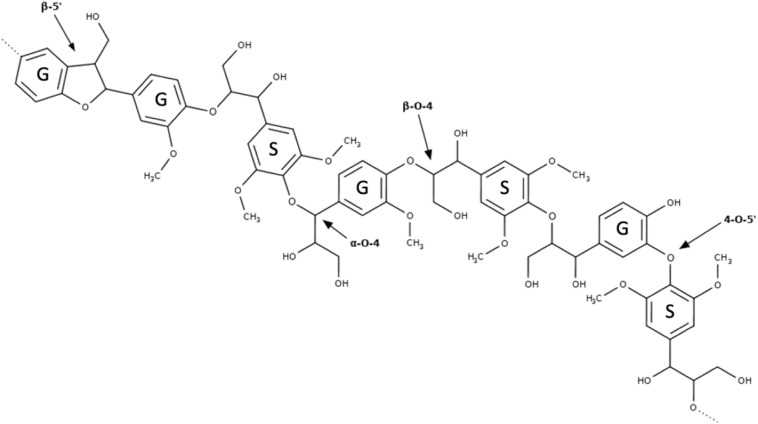
Hypothetical representation of a fragment arisen from a hardwood lignin macromolecular structure. G, guaiacyl units; S, syringyl units.

The reactivity of lignin relies mainly on its hydroxyl groups, phenolic or aliphatic, plus smaller amounts of carboxyl, and carbonyl groups (Figure 3). Therefore, lignin can be a source of several aromatic compounds and building blocks, essentially phenolics (Duval and Lawoko, 2014). However, regardless of its source, the structure of this macromolecule in its native form is still uncertain once studies indicated structural alterations when it is isolated from biomass (Pandey and Kim, 2011).

Lignin removal can beneficial for enzymatic activity through different mechanisms, since the high hydrophobicity of aromatic rings, hydroxylic aliphatic groups and low amounts of carboxylic groups can be factors that cause non-selective adsorption enzyme (Li et al., 2018). Chang and Holtzapple (2000) indicated that lignin removal is beneficial to hydrolysis because, besides being a physical barrier to cellulose access, the presence of lignin may give rise to compounds that are toxic to fermentative microorganisms. However, reports as Haven and Jørgensen (2013) and Jung et al. (2020) demonstrated that the presence of β-glucosidases reduces the lignin-related inhibitory effect in cellulase preparations composed by endoglucanases and cellobiohydrolases. This augment in cellulase activity could be explained by a significant β-glucosidase binding to lignin, preventing its inhibitory effect on cellulases. In fact, novel cellulase formulations (e.g., Cellic CTec3 from Novozymes) that are specific for hydrolysis of pretreated cellulosic materials possess high titles of β-glucosidase activity. Despite the reduction of lignin-related inhibitory effects by boosting the β-glucosidase component in cellulase preparations, current kinetic models normally based on lignin-free cellulosic materials.

## Pretreatment

Biomass pretreatment aims at fractionating lignocellulosic materials into useful streams for subsequent chemical or biological conversion. Due to cellulose crystallinity, matrix heterogeneity, low surface accessibility and lignin shielding, untreated lignocellulosic materials are highly recalcitrant, and this is expressed by their low convertibility to fermentable sugars by enzymatic hydrolysis, showing mass yields seldomly higher than 20% (Lynd et al., 2002). By contrast, pretreated materials generally exhibit increased porosity, high surface area and different levels of hemicelluloses and lignin removal, all of these very important to develop accessibility to cellulolytic enzymes and other conversion processes.

Table 1 describes the most widely studied techniques for pretreating and fractionating lignocellulosic materials. Biological, physical, chemical and hybrid (combined) processes are compared in relation to their mode of action. Biological pretreatments are very specific but usually time-consuming, working primarily for biomass conditioning prior to the application of a second pretreatment stage using acid, alkaline or oxidative reagents or catalysts. Physical pretreatments are energy intense if used alone and work best for biomass preparation to subsequent processing steps by decreasing particle size with the concomitant increase surface area, therefore facilitating biomass handling, stirring and impregnation with chemicals. Chemical pretreatments are addressed to the removal of plant cell components from low (extractives) to high molar mass (hemicelluloses, pectins and lignin), leaving a cellulosic slurry with high accessibility to the enzymatic hydrolysis. Finally, hybrid pretreatment techniques are normally the most efficient ones because they are based on the combination of two or more of the above. Examples for such strategy are (a) partial acid hydrolysis followed by alkaline delignification, (b) solvent extraction followed by acid hydrolysis, (c) milling followed by oxidative alkaline delignification; (d) milling followed by acid hydrolysis and/or alkaline delignification, and (e) microwave or ultrasound irradiation followed by acid/alkaline treatments, among other possibilities. Hybrid processes such as these can be based on a sequence of unit operations, but there are cases in which a good synergism is obtained by performing more than one pretreatment strategy simultaneously. However, in all of these situations, for isolated or combined pretreatment techniques, process optimization is critical to achieve high yields of process streams with good properties for their subsequent conversion to economically attractive products whose markets are in high demand for sustainability and process efficiency. Needless to say, this has a tremendous impact on the economic viability of the proposed pretreatment process, as well as in the demonstration of its compliance to regulatory rules and social demands.

**TABLE 1:** Description of the most relevant pretreatment techniques for assisting biomass conversion processes.

Process		Description
**Biological**	Fungi	Lignin biodegradation by laccases and manganese peroxidases. High selectivity at very long pretreatment times
	Bacteria	Genetically modified organisms that are able to convert biomass into fuels and chemicals (consolidated bioprocessing)
	Enzymes	Selective removal of high molar mass components in cellulosic matrices using lipases, lignin-degrading enzymes and hydrolases

**Physical**	Milling	Reduction of particle size and increase in substrate surface area for biological or chemical conversion processes
	Microwave	Disruption and swelling facilitating hemicelluloses and lignin removal; heating and reaction times are greatly reduced
	Ultrasound	Structure modification by cavitation; bonds in lignin-carbohydrate complexes are cleaved by radical chemistry

**Chemical**	Concentrated acid hydrolysis	Cellulose swelling and partial hemicellulose hydrolysis; lignin coalescence and condensation
	Dilute acid hydrolysis	Cellulose accessibility increased by partial hemicellulose removal; lignin coalescence, fragmentation, and condensation
	Alkaline extraction	Lignin extraction and partial hydrolysis of aryl-ether bonds, reducing its average molar mass
	Oxidation	Delignification with strong oxidants such as hypochlorite, oxygen radicals, and ozone
	Ionic liquids	Carbohydrate or lignin extraction due to its high polarity and strong intermolecular interactions with the cellulosic matrix
	Supercritical CO_2_	Partial acid hydrolysis of hemicelluloses; increase in substrate pore volume and available surface area

**Combined**	Organosolv	Selective biomass delignification, whose efficiency can be increased by adding an exogenous acid catalyst
	Kraft pulping	Alkaline delignification of lignocellulose at ∼170°C using aqueous Na_2_S/NaOH to isolate cellulose fibers (holocellulose)
	Sulfite pulping	Acid delignification at around 160°C using sulfite/bisulfite species to isolate almost pure cellulose fibers and lignin as lignosulfonate
	SPORL	Sulfite Pretreatment to Overcome Recalcitrance of Lignocellulose, developed from sulfite pulping to improve enzymatic hydrolysis
	AFEX	Partial removal of hemicelluloses and lignin plus changes in the crystalline state of cellulose (from I to III)
	Hydrothermal	Hemicellulose removal and lignin fragmentation and redistribution by autohydrolysis, increasing cellulose accessibility to enzymatic hydrolysis and hemicellulose recovery mostly as water-soluble oligosaccharides
	Liquid hot water	
	Steam explosion	Acid hydrolysis of hemicelluloses and lignin modification and redistribution; may be assisted by acid or basic catalysts

**Reprinted from Ramos et al. (2019) with permission from the Bulgarian Chemical Communications (Open Access).**

The level of chemical modification of lignocellulosic materials depends on the chemical properties of the reaction environment, which may promote hemicellulose removal and lignin oligomerization by acid hydrolysis, or lignin and/or hemicellulose extraction by alkali, organic solvents or bleaching agents. In most cases, glucans (mostly cellulose) must be recovered in high yields for further processing, while some hemicellulose sugars and lignin components may be partially lost in liquid or gaseous (air-born volatiles) pretreatment streams. Such mass losses normally translate into the accumulation of hydrolysis and/or fermentation inhibitors that are highly influential for bioconversion processes such as cellulosic ethanol production. Furans, organic acids, phenolic acids and oligomeric components build up in the system according to pretreatment severity and, unless when identified as desired reaction products, their concentration in process streams must be minimized as much as possible.

Reducing the formation of inhibitors is a critical aspect for an optimal pretreatment process because detoxification strategies can be costly and lead to high sugar losses. Furan compounds such as furfural and 5-HMF originate from dehydration of pentoses and hexoses at high temperatures, respectively (Jönsson and Martín, 2016). Weak acids such as acetic, levulinic and formic are released in biomass hydrolyzates, and these are also known as fermentation inhibitors. While acetic acid comes from hydrolysis of *O*-acetyl groups in hemicelluloses, levulinic and formic acids originate from degradation of 5-HMF at more severe pretreatment conditions (Palmqvist and Hahn-Hägerdal, 2000). Lignin degradation and solubilization under high temperature and pressure conditions may lead also to the release of phenolic compounds that inhibit enzymatic activity such as vanillin, syringaldehyde, 4-hydroxybenzoic acid, trans-cinnamic acid, *p*-coumaric acid, and ferulic acid (dos Santos et al., 2019).

Rajan and Carrier (2014) studied the effect of dilute sulfuric acid concentration on wheat straw fractionation, as well as the impact of temperature on the release of fermentation inhibitors. High acid concentrations had a positive effect on the release of formic and acetic acids, as well as furfural and 5-HMF. An increase in sulfuric acid concentration between 5 and 20 dm^3^⋅m^–3^ led to a decrease of up to 27% in the production of monosaccharides. Xu et al. (2017) studied the pretreatment of eucalyptus by dilute acid hydrolysis and hydrothermolysis. Dilute acid pretreatment using 0.25 mol⋅L^–1^ H_2_SO_4_ and a liquid-to-solid ratio of 1:20 (g⋅mL^–1^) at 90°C for 1 h led to the formation of 3.26 g⋅L^–1^ levulinic acid, 4.26 mg⋅g^–1^ acetic acid, 1.24 g⋅L^–1^ 5-HMF and 0.86 g⋅L^–1^ furfural. In comparison, hydrothermolysis at 180°C for 30 min using deionized water in a solid ratio of 1:20 (g⋅mL^–1^) yielded 2.06 g⋅L^–1^ levulinic acid, 2.81 g⋅L^–1^ acetic acid, 1.93 g⋅L^–1^ furfural, and 2.07 g⋅L^–1^ 5-HMF.

Green solvents have emerged in the last few years as good alternatives for biomass pretreatment because their application does not require the use of severe pretreatment conditions. In this way, ionic liquids are regarded as a relevant class of green solvents that are capable of replacing several hazardous volatile organic solvents. A variety of ionic liquids (ionic compounds that are in liquid state at 100°C and ambient pressure) has been tested so far for biomass fractionation (Halder et al., 2019). The physicochemical properties of these specialty solvents include negligible vapor pressure and high thermal and chemical stabilities, as well as a high capacity to solubilize organic and inorganic, as well as polar and non-polar substances, depending on its chemical composition (Boruń, 2019). Swatloski et al. (2002) studied the solubility of cellulose in various ionic liquids, showing that the most effective were those containing anions which are strong hydrogen bond acceptors.

Pretreatments with ionic liquids do not cause lignocellulose degradation because they are usually carried out at relatively mild conditions to simply dissolve biomass fractions, either polysaccharides or lignin (Zhang et al., 2015). In the presence of antisolvents such as water, ethanol or methanol, the preferential solute displacement mechanism occurs, which is characterized by the instantaneous precipitation of the pre-dissolved cellulose (Swatloski et al., 2002; Dadi et al., 2006).

Although not yet economically viable for industrial applications due to their high costs, ionic liquids do not degrade polysaccharides and produce less inhibitors than conventional chemical treatments (Zhang et al., 2015). For this reason, the application of ionic liquids as co-solvent may be an economically viable choice to exploit their unique physical properties (Silveira et al., 2015b; Boruń, 2019). However, ionic liquids themselves may be detrimental to hydrolysis and fermentation. Therefore, for both practical and economic reasons, their recovery and reuse must be optimized for optimal performance.

## Supercritical Fluids

Supercritical fluids are substances above their critical conditions of temperature and pressure. At these conditions, a fluid does not present vapor-liquid phase transition, existing only in a homogeneous phase-condition whose properties such as diffusivity, viscosity and density lie between those of gases and liquids (Ibáñez et al., 2016). Also, supercritical fluids may have their properties tuned by adjusting pressure or temperature, or by combination with specific liquid solvents in function of effects entrained by chemical association between both modifiers and solutes (Walsh et al., 1987). Compounds that can be highlighted among the most common supercritical fluids are carbon dioxide, ammonia, water and hydrocarbons such as propane and butane.

Supercritical fluids can be applied for biomass valorization both as a pretreatment technique or as a reactive extraction procedure to yield value-added coproducts. The first aims to enhance substrate accessibility to enzymatic hydrolysis by causing a physical and/or chemical disruption of the lignocellulosic matrix. By contrast, the latter deals with direct carbohydrate hydrolysis and lignin transformation into liquid fuel and char, among others. Generally, increased temperatures and pressures enhance reaction performance by improving solvent penetration through enlarged fiber pores and defects. However, carbohydrate degradation may occur at high temperatures, generating furan derivatives and organic acids mostly by carbohydrate degradation (Luterbacher et al., 2010).

In general, the physical structure of biomass changes when subjected to a treatment involving solvents at high pressure conditions. Gas-expanded liquid solvents permeate cell wall micropores when biomass is exposed to high pressure. With the sudden depressurization of reaction chamber, the highly compressible fluid leaves the solution quickly causing a rapid expansion of the plant cell wall. This effect alters the biomass physical structure by increasing its fiber porosity and surface area. Hence, when high-pressure treatments are performed, particularly at high temperatures, good substrates for enzymatic hydrolysis are produced because the biomass structure is outstretched, allowing a subtle enhancement in enzyme-substrate interactions (Raud et al., 2016).

### Supercritical Water

Supercritical conditions of water are reached at 374°C and 221 bar. Above these conditions, the dielectric constant of water decreases, increasing the solubility of organic compounds in it. At 250 bar and between 300 and 400°C, its ionic product ranges from 10^–10^ to 10^–22^, enabling enhanced selectivity in chemical reactions and changing the ionic reaction mechanisms to free radicals. In addition to these improvements in physicochemical properties, there is no need to dry the biomass before treatment, and the resistance to mass transfer is reduced or even eliminated (Cantero et al., 2015). However, the use of this technique requires extremely low reaction times to avoid C_6_ (glucose) and C_5_ (xylose, arabinose, etc.) sugar degradation, limiting its application to ultra-fast reactors with residence times of only 1 s (Cantero et al., 2013).

The fast hydrolysis of wheat bran was evaluated by Cantero et al. (2015) at 400°C and 250 bar for 0.19 s, showing an average conversion of 73 wt.% of hemicelluloses and glucans (mostly cellulose) into C_5_ and C_6_ sugars with the release of only 0.5 wt.% in furfural and 5-HMF. The remaining solids after hydrolysis consisted of 85 wt.% lignin, with about 5 wt.% glucans still remaining in the hydrolysis residue. Martínez et al. (2018) studied the implementation of supercritical water for hydrolysis of beet pulp at 390°C and 250 bar, varying the reaction times between 0.11 and 1.15 s. The highest C_6_ and C_5_ yields of 61 and 71 wt.% were obtained at 0.11 s, respectively, and the use of short reaction times produced low concentrations of dehydration byproducts. Also, Martínez et al. (2019) compared the supercritical hydrolysis of sugar beet pulp and wheat bran at laboratorial and pilot scale and concluded that long hydrolysis times were detrimental to the obtainment of high sugar yields, however, in pilot scale tests, the use of higher average particle sizes reduced sugars degradation, leading to sugar yields up to 90% of the theoretical maximum.

Jeong et al. (2017) studied the hydrolysis of *Quercus mongolica* carbohydrates in a pilot scale unit using supercritical water in the presence of an acid catalyst. Hydrolysis was carried out at 380°C and 230 bar using 0.01–0.10 wt.% H_2_SO_4_, a solid to liquid ratio of 1:50 and reaction times below 1 s. The best performance was obtained with 0.05% H_2_SO_4_, which enhanced sugars yields from 19.7 wt.% (non-catalyzed) to 35.3 wt.% (pretreated biomass). The production of fermentation inhibitors (furfural, 5-HMF and acetic, formic and levulinic acids) remained almost constant between non-catalyzed and acid-catalyzed conditions using 0.05% H_2_SO_4_, but their presence increased when the acid concentration was raised to 0.10% H_2_SO_4_. Moreover, with the use of an acid catalyst, acid hydrolyzates had to be detoxified prior to fermentation.

Harnessing agro-industrial wastes for hydrogen power generation is a sustainable way to add value to otherwise worthless materials and help the environment by reducing greenhouse gas emissions (Ferreira-Pinto et al., 2019). Gasification is an oxidative thermochemical process that aims at the conversion of biomass into fuels (mostly syngas), hydrogen and methane, among others (Farzad et al., 2016). Various agents may be applied for biomass gasification, such as air, steam or oxygen to produce low, medium and high calorific value syngas, respectively (Ruiz et al., 2013). Due to high cost of oxygen, alternative agents have been investigated and one of the most promising is water at subcritical or supercritical states. Biomass gasification in water is a very complex endothermic process where water, besides being solvent, is also the reagent from which hydrogen is obtained (Guo et al., 2007). The reaction can be summarized by the following equation:

CHOx+y(2-y)HO2→CO+2(2-y+x/2)H2

Upon optimization of pretreatment variables such as temperature and pressure, supercritical water may provide a rapid process for biomass gasification (Cocero et al., 2018). Under supercritical conditions, water undergoes changes in properties such as its dielectric constant, which becomes similar to that of an organic solvent. Gases are also solubilized under these conditions, leading to a single phase in which a reaction cascade involving pre-solubilized compounds may take place (Withag et al., 2012). Also, the lower viscosity of supercritical water enables a better diffusion into the matrix, facilitating the reactive extraction and/or solubilization of organic compounds. Guo et al. (2007) compared several biomass gasification studies and concluded that temperature plays a very important role in biomass gasification, particularly in the absence of an added catalyst. In general, optimal biomass gasification is achieved at temperatures ranging from 650 to 800°C.

According to Correa and Kruse (2018), hydrothermal gasification can be divided in three categories according to Table 2. In treatments performed at temperatures above the water supercritical point, cellulose hydrolysis occurs not only at the terminal chain ends, but also randomly at the middle of the chains, increasing its solubilization in water. This way, a two-stage thermal treatment is possible by first subjecting the plant biomass to temperatures above the supercritical condition for cellulose solubilization, followed by exposure to subcritical conditions for optimal hydrolysis to monosaccharides and low molar mass oligomers (Correa and Kruse, 2018).

**TABLE 2:** General products obtained by pressurized water treatment.

Category	Temperature (°C)	Products
Aqueous phasing reforming	215–265	H_2_ and CO_2_
Near critical gasification	350–400	CH_4_
Supercritical water gasification	>500	H_2_ and CO_2_

Biomasses with high lignin contents generate lower hydrogen yields by supercritical water gasification. Under supercritical conditions, lignin is fragmented into low molar mass phenolic compounds and other species such as reactive aldehydes that may condense into undesirable recalcitrant by-products and contribute to the buildup of residual char (Madenoglu et al., 2011).

Catalysts are useful adjuvants to reduce process costs by decreasing the temperature and pressure required for optimal biomass gasification. It is also desirable that catalysts could prevent polymerization of intermediate compounds such as furans, aromatic aldehydes and phenols, therefore improving gasification yields. Examples of catalysts are included in the classes of alkali metals, transition metals, metal oxides and minerals (Ferreira-Pinto et al., 2019).

Madenoglu et al. (2011) studied the supercritical water gasification of various agricultural residues such as cauliflower, tomato peel, and hazelnut shells at 600°C and 350 bar for 0.3 min, with and without addition of K_2_CO_3_ as catalyst. Materials treated without an added catalyst had a hydrogen production of 20.2, 17.9, and 11.7 mol⋅kg^–1^ (dry basis), while these values increased to 32.1, 30.9, and 18.5 mol⋅kg^–1^ when K_2_CO_3_ was added to the production process, respectively.

Lu et al. (2008) treated a mixture containing 8% corn cobs and 2% sodium carboxymethylcellulose (CMC) in a fluidized bed reactor at 600°C and 250 bar. Gases were obtained in molar ratios of 37% for H_2_, 3% for CO, 8% for CH_4_, 48% for CO_2_ and less than 3% for C_2_H_4_ and C_2_H_6_. Increasing the feedstock concentrations reduced H_2_ yield and variations in pressure resulted in scattered responses, either positive or negative. Therefore, further investigation related to gasification in this type of reactor is still in need for further process optimization. In a similar study, Lu et al. (2006) treated 2% sawdust with 2% CMC in a supercritical fluid reactor at 650°C and 30 MPa for 27 s. Gasification efficiency was approximately 100% with an H_2_ production of approximately 17 mol⋅kg^–1^. Under the same pressure and reaction time, a reduction of 50°C in temperature reduced H_2_ production by approximately 3 mol⋅kg^–1^, leading to an ∼85% gasification efficiency. From this and other studies, it is evident that temperature exerts a very high influence on biomass gasification.

### Supercritical CO_2_ Pretreatment

Supercritical carbon dioxide (scCO_2_) is one of the most used compressed fluids for biomass processing due to its moderate critical conditions (31.1°C and 74 bar), non-flammability, low-toxicity and wide availability. Traditionally described as a non-polar molecule because of its zero-dipole moment, scCO_2_ presents its maximum solvation power for non-polar or weakly polar compounds, which is inversely proportional to the molar mass of the solute (Brunner, 2005). However, CO_2_ presents a significant quadrupole moment, and, related to its microscopic solvent behavior, this molecule may participate in hydrogen-bond interactions and act as both weak Lewis’ acid and base (Raveendran et al., 2005). Furthermore, it may have its solvation power altered by the addition of co-solvents, which can increase the solvent system polarity. Besides that, scCO_2_ facilitates mass transfer by enhancing diffusivity and lowering viscosity of the solvent system. Additionally, the moisture present in the biomass together with CO_2_ generates carbonic acid, which can promote hemicellulose hydrolysis (Narayanaswamy et al., 2011; Daza Serna et al., 2016; Fockink et al., 2018). One of the main economic advantages of scCO_2_ pretreatment is that no fermentation inhibitors are produced; hence, once the extraction is over, the biomass is ready to be hydrolyzed and subsequently fermented without the need for detoxification or any separation/purification process (Gu et al., 2013).

Zheng et al. (1995) were pioneers in reporting the use of scCO_2_ as a pretreatment strategy for cellulosic materials. Microcrystalline cellulose (Avicel) was pretreated with CO_2_ in subcritical and supercritical states at mild temperature conditions (35–80°C and 69–276 bar) to improve its susceptibility to enzymatic hydrolysis. Later, Zheng et al. (1998) studied the pretreatment of other materials (recycled paper and sugarcane bagasse) with subcritical CO_2_ and scCO_2_ at temperatures between 25 and 80°C and pressures from 76 to 276 bar. These authors found differences in glucose yield after enzymatic hydrolysis between both pretreatment strategies, with the best results obtained at the highest pressure and temperature where yields were increased by as much as 50%. However, the yield increase caused by subcritical CO_2_ was neglectable compared to the untreated material, which may happen due to the poor diffusibility of liquefied CO_2_ into the micropores of the cellulose structure.

The effect of biomass moisture content on scCO_2_ have been extensively studied due to the possible formation of carbonic acid, which hydrolyzes hemicelluloses and enhances substrate accessibility to enzymatic hydrolysis. Kim and Hong (2001) evaluated the effect of moisture on scCO_2_ pretreatment of aspen and southern yellow pine (SYP) at 165°C and 214 bar. Moisture content variations from zero to 73% presented a positive effect over sugar yields after pretreatment and enzymatic hydrolysis. Reducing sugar yields were enhanced from 14.5% for the untreated aspen biomass to 84.7% after scCO_2_ pretreatment. However, at the same conditions, SYP pretreatment presented a much lower yield increase, from 12.8% for the untreated biomass to 27.3% after scCO_2_ pretreatment and this was attributed to the lower tissue porosity and higher recalcitrance of softwood guaiacyl lignin.

Attard et al. (2016) studied the scCO_2_ wax extraction of miscanthus as a pretreatment strategy. At the best condition (50°C and 350 bar, giving a 1.58 wt.% wax extraction yield), pretreatment enhanced enzymatic hydrolysis in 20% compared to the untreated material. Srinivasan and Ju (2010) evaluated the pretreatment of guayule bagasse (a high lignin content biomass) with moisture contents between 50 and 75% using scCO_2_ and compared the sugar conversion with dilute acid hydrolysis. Total reducing sugars yields of 86% (scCO_2_ at 200°C and 276 bar for 60 min) and 52% (dilute acid at 10 wt.% total solids and 0.75% H_2_SO_4_ at 180°C for 5 min) in relation to the theoretical maximum, respectively. Additionally, these authors reported the presence of inhibitors in the acid pretreatment liquor, which was consistent with the observation of higher mass losses; by contrast, no solid loss was detected after scCO_2_. Narayanaswamy et al. (2011) studied the pretreatment of switchgrass and corn stover with scCO_2_ followed by rapidly depressurization. For corn stover, pretreatment of dry matter led to a neglectable increase in glucose yield, whereas for biomass with a 75% moisture content, the yield increased from 12 to 30% at 150°C and 241 bar. However, under similar experimental conditions using switchgrass, glucose yields did not show a significant increase after pretreatment (from 12 to 14%). Besides, no effect was found of scCO_2_ on crystallinity index (through X-ray diffractometry, XRD), while scanning electron microscopy (SEM) images indicated an increase in surface area.

The effect of extended scCO_2_ pretreatment times under mild temperature conditions was studied by Zhao et al. (2019). Corn stover, corn cob and sorghum stalk with 75% moisture contents were pretreated at temperatures between 50 and 80°C, pressures between 175 and 250 bar, and reaction times from 12 to 60 h. After enzymatic hydrolysis, untreated corn stover, corn cobs and sorghum stalk yielded 16, 14.8, and 12.7% in simple sugars in relation to the theoretical maximum, but after scCO_2_ sugar yields increased to 62.2% (70°C, 225 bar and 48 h), 45.6% (70°C, 200 bar and 48 h), and 47.2% (80°C, 250 bar and 24 h), respectively. These authors claimed that longer pretreatment times favored biomass swelling in water and aided scCO_2_ penetration into the lignocellulose microstructure. During the depressurization step, scCO_2_ destroyed the structure of the cell wall and turned the native biomass more susceptible to hydrolysis.

Another technique for biomass pretreatment employs mixtures of scCO_2_ and water. Due to the insufficient capability of water to dissolve CO_2_, a biphasic system is formed, but the acidity of the medium is altered, lowering its pH (Jessop and Subramaniam, 2007). Luterbacher et al. (2010) applied CO_2_ + H_2_O at high total solids to various lignocellulosic matrices, with pretreatment time varying from 20 s to 60 min and temperatures between 150 and 200°C, while pressure was maintained constant at 200 bar. At the best conditions (170°C and 60 min), mixed hardwoods presented theoretical glucan yields rising from 5.1 to 73% using 20 and 40 wt.% total solids, respectively. Also, sugar dehydration to furfural and 5-HMF increased 19 and 5% at temperatures between 150 and 250°C. Later, Luterbacher et al. (2012) applied CO_2_ + H_2_O in a two-temperatures sequential process: one high temperature/short time stage (210°C, 200 bar for 16 min) followed by one low temperature/long time stage (160°C, 200 bar for 60 min). This strategy was performed at 40 wt.% total solids using particle sizes 10 times larger than those used in their previous work and, despite that, glucose yields from mixed hardwoods reached 83% of the theoretical maximum.

Pretreatment with scCO_2_ seems to produce a physical effect that is due to the rapid expansion of CO_2_ inside the fiber structure. Benazzi et al. (2013) studied the pretreatment of sugarcane bagasse (45–65% moisture) using scCO_2_ at 40–80°C, pressures between 100 and 250 bar, and reaction times of 30–120 min. The rate of depressurization to atmospheric, ranged from 50 to 200 kg⋅m^–3^⋅min^–1^, had no significant effect on substrate accessibility to enzymatic hydrolysis. The effect of physical explosion was also investigated by Islam et al. (2017) for the scCO_2_ pretreatment of soybean hulls with a 66.7% moisture content at temperatures between 80 and 180°C and pressures from 52 to 124 bar. The use of rapid rather than controlled depressurization improved total reducing sugar yields by 20% when pretreatment was carried out at 180°C and 86 bar.

The chemical and physical effects of scCO_2_ may be enhanced by applying multiple stage pretreatment strategies. Phan and Tan (2014) studied the synergistic effects between scCO_2_ (180°C, 206 bar for 60 min) and alkaline H_2_O_2_ or ultrasound irradiation, with both performed at pH 11.5 due to the continuous addition of aqueous sodium hydroxide to the reaction medium. An almost theoretical glucose recovery of 97.8% was obtained using scCO_2_ followed by alkaline H_2_O_2_ (60°C and 0.6% H_2_O_2_ for 9 h). This yield was higher than those obtained by applying scCO_2_ (61.3%) or alkaline H_2_O_2_ (22.9%) alone, all of these excelling the value obtained from the untreated biomass (13.4%). By contrast, when scCO_2_ was followed by ultrasound irradiation at pH 11.5, the glucose recovery (65.8%) was close to that of scCO_2_ alone. The enhanced performance of scCO_2_ followed alkaline H_2_O_2_ may be related to the decomposition of H_2_O_2_ in alkaline medium, which generates hydroxyl (HO^–^) and superoxide (O_2_^–^) anion radicals that are capable of oxidizing lignin and promoting higher levels of alkaline delignification.

The use of ultrasound followed by scCO_2_ was investigated by Yin et al. (2014) for the pretreatment of corn stalks and corn cobs. When scCO_2_ was carried out alone at 120–170°C and 150–250 bar for 30 min, followed by rapid depressurization, the use of high temperatures and intermediate pressures (200 bar) had a positive effect on pretreatment efficiency. Then, ultrasonication was applied prior to scCO_2_ by soaking the biomass in deionized water for 24 h and exposing it to cavitation (20 kHz, 600 W) at 80°C for 2–8 h; afterward, the biomass was drained up to a 50% moisture content and subjected to scCO_2_ at 170°C and 200 bar. Ultrasonication for no longer than 6 h seemed to enhance the total reducing sugar yields from corn cobs. While the untreated biomass yielded 12.5%, such yields rose to 62% after scCO_2_ and to 87% after ultrasound followed by scCO_2_. By contrast, pretreatment of corn stalks suffered minor changes due to the use of both ultrasound and scCO_2_, yielding 16.6% in total reducing sugars for the untreated biomass, 25.5% after scCO_2_ and 30% after ultrasound followed by scCO_2_. As reported by previous studies, no change in crystallinity was observed between untreated and pretreated materials by XRD analysis, while SEM images revealed ruptures on the fiber surface, with greater disruption levels being observed after ultrasound followed by scCO_2_.

Steam explosion followed by scCO_2_ was proposed by Alinia et al. (2010) for the pretreatment both dry and wet (23% of moisture) wheat straw and the results were compared to those of scCO_2_ pretreatment alone. Steam explosion was carried out at 200 and 210°C for 10 and 15 min, whereas scCO_2_ was performed at 160 and 200°C for 10, 30, and 60 min. After enzymatic hydrolysis, scCO_2_-pretreated dry wheat straw yielded 14.9 wt.% in reducing sugars, while the wet biomass increased this yield to 20.9 wt.%. The two-stage process, however, enhanced reducing sugar yields by 12% to 23.5 wt.%. The poor performance of this later strategy might have been due to the harsh conditions used for steam explosion, which overshadowed the possible benefits of scCO_2_ by removing hemicelluloses almost completely and redistributing the lignin component in such a way to produce substrates with high accessibility to enzymatic hydrolysis.

Santos et al. (2011) investigated the alkaline delignification of sugarcane bagasse and compared it with a two-stage pretreatment process using alkaline delignification followed by scCO_2_. In the first stage, the biomass was cooked in a caustic solution (0.20 g NaOH⋅g^–1^ of bagasse in 100 mL of distilled water at 100°C for 60 min), washed with distilled water until neutrality and dried at 37°C. Finally, at the second stage, scCO_2_ was applied at 160 bar for 60 min. The use of scCO_2_ after alkaline delignification increased glucose yields by 20%, compared to alkali delignification alone.

The addition of co-solvents in scCO_2_ processes is a promising alternative to alter solvent system properties, modifying polarity and solubility parameters. The effect of co-solvent addition in the extraction of vegetable matrices are well elucidated and mainly related to an increase in solvent polarity (Melo et al., 2014). Pretreatment of palm empty fruit bunches (EFB) using scCO_2_ under alkaline conditions was evaluated and compared to the use of scCO_2_ alone at different temperatures (80–130°C), pressures (150–250 bar) and residence times (30 or 60 min). For the alkali + scCO_2_ pretreatment, the matrix was premixed with 4% aqueous sodium hydroxide to reach a 75% moisture content (0.12 g NaOH⋅g^–1^ dry EFB), and subsequently exposed to scCO_2_ at 80°C and 250 bar for 30 min. The untreated EFB yielded 17 wt.% in glucose (dry basis), while scCO_2_ yielded 24 wt.% when operated at the upper limit of the pretreatment variables. However, EFB pre-impregnation with alkali resulted in an almost theoretical glucose yield, but its effect on biomass delignification was neglectable probably due to the low NaOH-to-EFB employed in this study (Hamzah et al., 2015).

Rosero-Henao et al. (2019) investigated the use of alkaline soaking followed by compressed CO_2_ to improve the methane production from sugarcane bagasse by anaerobic digestion. These authors evaluated near critical state CO_2_ (40°C, 69 bar) and scCO_2_ (60 and 80°C, 196 bar) with and without premixing the biomass with an aqueous sodium hydroxide solution (2 g⋅L^–1^). In the absence of alkaline pre-soaking, scCO_2_ at 60°C led to the highest delignification extent (8.1%) and to a methane production that exceeded the yield obtained from the untreated material by 23.4%. Compared to scCO_2_ alone, the use of alkali followed by scCO_2_ had a slightly negative effect over methane production, even enhancing the lignin content in pretreated biomass. This effect might have been related to the low temperatures employed in this study, in which lignin may have been partly dissolved, but no further eliminated nor diffused out of the fiber network. Also, fiber hornification may have occurred because pretreated materials were dried prior to anaerobic digestion.

Organosolv processes stand out as promising alternatives for the optimal pretreatment of lignocellulosic materials and have been extensively investigated for biomass valorization (Choi et al., 2019; Tan et al., 2019; Cebreiros et al., 2020). However, pretreatment efficiency may be enhanced by adding scCO_2_ in a typical CO_2_-expanded liquid (CXL) scheme. At this condition, organic solvents have the ability to dissolve large amounts of CO_2_, expanding greatly and altering physical properties such as diffusivity, viscosity and surface tension: the first is increased while the other two are dramatically decreased in the presence of CO_2_ (Jessop and Subramaniam, 2007; Corazza et al., 2019). Pasquini et al. (2005b) investigated the delignification of sugarcane bagasse using 1-butanol + water with scCO_2_ in CXL scheme. The 1-butanol content in water varied from 60 to 90%, the temperature between 150 and 190°C, and the pressure between 70 and 230 bar. These authors found that the lowest alcohol content at the highest pretreatment temperature led to the highest delignification extent (94.5%), but selectivity for lignin was low, leading to an extensive polysaccharide mass loss (8.7 wt.% pulp yield). Also, Pasquini et al. (2005a) applied ethanol + water mixtures under scCO_2_ for the delignification of sugarcane bagasse and *Pinus taeda* wood chips. The co-solvent composition was evaluated from 50 to 100% ethanol for SCB and from 30 to 100% for pine wood chips. Both solvent nucleophilicity (for lignin cleavage) and lignin solubility were identified as important factors, with the highest delignification extent being obtained with 50% ethanol for both lignocellulosic matrices. The highest delignification extents were 88.4% for SCB and 93.1% for pine wood chips at 190°C and 160 bar, but the pulp yields were relatively low at 32.7 and 43.7 wt.%, respectively.

Lü et al. (2013) studied the scCO_2_ pretreatment of corn stover in combination with water, ethanol and water/ethanol mixtures to improve of enzymatic hydrolysis of cellulose. Hydrolysis of the untreated biomass yielded 8.5 wt.% in total sugars, whereas scCO_2_ increased it to 23.3 wt.%. The use of water (20 mL) or ethanol (300 mL) with scCO_2_ raised these total sugar yields to 38 and 37 wt.%, respectively, suggesting that ethanol is not the ideal co-solvent to improve biomass accessibility to hydrolysis. Thus, to improve pretreatment performance, mixtures of water and ethanol were use in the presence of scCO_2_ and, at the best experimental condition of 180°C and 150 bar for 60 min using a water-to-ethanol volumetric ratio of 2:1, 77.8% of the theoretical sugars were obtained after enzymatic hydrolysis. In another study, the influences of pretreatment temperature (160–200°C), pressure (130–170 bar) and time (40–80 min) in scCO_2_ + H_2_O + EtOH systems were evaluated for lignin removal from corn stover. The highest delignification extent (90.0%) was obtained in the upper levels of temperature and time. However, the highest glucose conversion (80.5%) was obtained with a delignification extent of 83.6% (180°C and 130 bar for 60 min) because pretreatment at 200°C, despite increasing delignification, led to relatively high sugar losses. Lignin droplets were observed by SEM on the fiber surface, but a final washing step using aqueous ethanol (2:1) removed this coalesced lignin fragments, enhancing glucose yields from 80.5 to 92.0% after enzymatic hydrolysis (Lv et al., 2013).

Daza Serna et al. (2016) investigated the effects of scCO_2_ + H_2_O + EtOH on rice husks delignification at 80°C and 270 bar for 10 min using a water-to-ethanol volumetric ratio of 2:1. For comparison effects, a dilute-acid pretreatment was performed at 25% total solids using 2% H_2_SO_4_ at 120°C for 1 h and a solid-to-liquid ratio of 1:10. At the best experimental condition, biomass delignification reached 90.6% and enzymatic hydrolysis yielded 7.16% total reducing sugars in the absence of any β-glucosidase activity (using only endoglucanases and exoglucanases), compared to 6.5% for dilute acid pretreatment yielded and 4.11% for untreated rice husks. Although indicative of some favorable effect on accessibility, these modest improvements in glucose yield may be associated to the dehydrating effects of ethanol causing substrate hornification after pretreatment.

The delignification of lignin model compounds during organosolv pulping was evaluated by Schrems et al. (2012) in the absence and presence of scCO_2_. Addition of scCO_2_ seemed to lower the activation energy for delignification in about 40%. Hence, the effects of scCO_2_, besides reducing mass transfer resistance and enhancing substrate accessibility to hydrolysis, changed chemical pathways by altering solvent polarity and the kinetics of organosolv pulping.

Silveira et al. (2015b) evaluated the effect of low concentrations of 1-butyl-3-methylimidazolium acetate ([Bmin][OAc]) in the scCO_2_ + ethanol pretreatment of extractives-free sugarcane bagasse (SCB). Pretreatment was carried out using 20 mL ethanol and 1–2 mL of the ionic liquid (IL) in a 50 mL agitated Parr reactor containing 2 g of SCB. The variables evaluated were temperature (110–180°C), pressure (195–250 bar), and IL-to-SCB ratios (0:1–1:1, vol⋅wt^–1^). At the best pretreatment condition (180°C, 250 bar and IL-to-SCB ratio of 1:1), the delignification extent was maximized in 42% and the glucose yield using low enzyme loadings of Cellic CTec2 (Novozymes) reached 70.7 wt.% of the theoretical maximum. High selectivity for delignification was not a requirement to develop high susceptibilities to enzymatic hydrolysis, with emphasis to the small amount of IL applied for optimal SCB pretreatment. Generally, the amount of IL employed for biomass fractionation is at least 10 times higher than the IL loading used in this study (Tan et al., 2009; Verdía et al., 2014).

### Other Supercritical Fluid Technologies

Alternative supercritical fluid systems can be applied for biomass fractionation into value-added co-products. Supercritical ammonia was studied by Bludworth and Knopf (1993) for the pretreatment of yellow poplar using water as co-solvent (zero to 20%), temperatures from 160 to 200°C, pressures from 138 to 276 bar and up to 3 h reaction times. At the best condition (20% water, 200°C and 207 bar), delignification reached 70% and glucose yields after enzymatic hydrolysis were 73 wt.% of the theoretical maximum. A power-law based modeling equation was presented with near first order dependence with ammonia and negative order dependence with water. However, water showed itself important to improve selective delignification, while neat ammonia resulted in substrates with lignin contents similar to those of the untreated biomass. Recently, Zhang et al. (2019) studied bamboo pulping using supercritical ammonia at 185 and 190°C and compared the pretreated material with conventional *kraft* pulping. Differences were reported with regard to differential thermal analysis, scanning electron microscopy and X-ray diffractometry for both pretreatment techniques. In general, the bamboo pulps produced by supercritical ammonia met all quality requirements for paper making.

Another process for biomass conversion under supercritical conditions is the production of bio-oil through direct liquefaction of lignocellulosic materials. Bio-oil was produced from rice stalk using two torrefaction steps (temperatures from 200 to 280°C) followed by liquefaction in supercritical ethanol (325°C and 140–150 bar for 60 min), and the results were compared to the direct liquefaction of the same matrix under otherwise identical experimental conditions (Li et al., 2014). Bio-oil yields were reduced from 55.0% for the direct liquefaction of rice stalk to 49.8 and 38.6% for the liquefaction of materials torrefied at 200 and 280°C, respectively. Also, the solid residue increased from 21.7% for direct liquefaction to 26.6 and 40.3% for the torrefaction/liquefaction process, respectively. On the other hand, the bio-oil properties were enhanced by previous torrefaction, being best when this process was carried out at 200°C. Bio-olis obtained in this way presented high ester contents, low acid values, and the highest heating value of 32.53 MJ⋅kg^–1^.

Selective lignin depolymerization (SLD) of sorghum bagasse was studied by Sagues et al. (2018) using subcritical (180°C) and supercritical ethanol (250°C) for 0.5–3 h with previous Fenton oxidation using 20% H_2_O_2_ at 60°C for 1 h. Supercritical ethanol provided higher lignin depolymerization and better holocellulose preservation compared to subcritical ethanol. Besides that, within 3 h of SLD, supercritical ethanol enhanced the production of phenolic oils by 145% compared to subcritical ethanol. It was hypothesized that ethanol at its critical stage may have increased its reactivity with lignin due to its non-polar nature, with the concomitant decrease in reactivity toward cellulose and hemicelluloses. Also, 66 wt.% C_5_ + C_6_ sugars (dry basis) were obtained by enzymatic hydrolysis of cellulosic materials that were obtained after performing SLD for 1 h.

## Further Valorization of Biomass Compounds: a Biorefinery Approach

**FIGURE 4:**
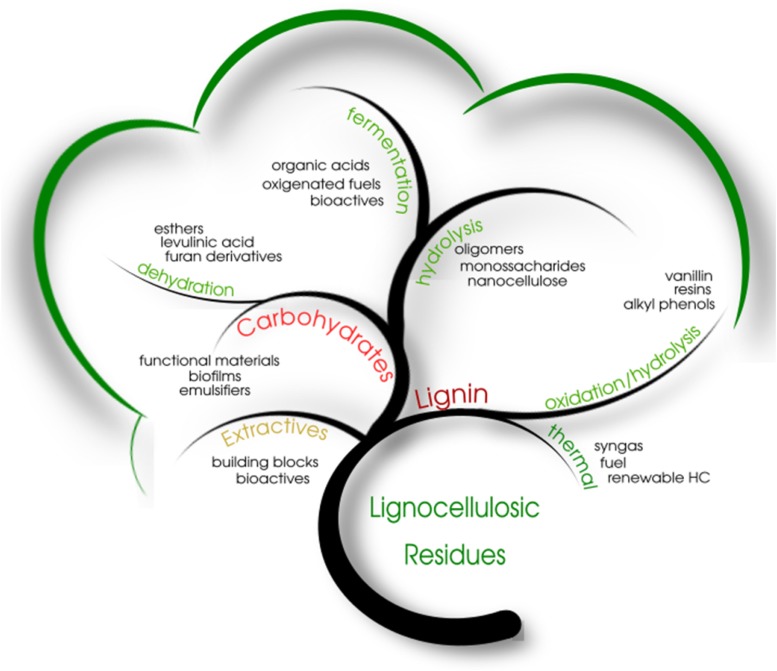
Lignocellulosic constituents and their products.

Figure 4 depicts some of the most prominent products that can be derived from each of the main constituents of lignocellulosic materials under a biorefinery concept. This concept may be seen as the sustainable use of biomass to produce energy and marketable products. A myriad of studies has been done so far on biomass conversion processes to produce value-added chemicals, fuels and biomaterials, aiming to promote its complete utilization without generating environmentally hazardous wastes including air-born emissions (Cherubini, 2010). After an appropriate single- or multi-stage pretreatment process, lignocellulosic materials have their constituents separated and processed, leading to the production of commercially interesting products. This approach tries to reach out to the full utilization of these process streams in order to enhance the economic viability of the entire production chain. In general, a single processing route is not sufficient to achieve economic viability, and potential wastes are inevitably discarded. Thus, it is necessary to look at lignocellulosic materials in a holistic way, seeking as much as possible for process integration and process intensification.

One reasonable strategy for the optimal utilization of biomass is the pre-extraction of oils, waxes, carotenoids, sugars, phenolics and other compounds that can be used directly or as a precursor for a variety of chemicals with a wide range of applications in cosmetics and in the medical and food industries (Reverchon and de Marco, 2006). In many situations, a simple procedure is enough to extract these low molar mass compounds for further use as surfactants, food additives, antioxidants, chemical precursors, bioactive compounds and building blocks for biocompatible polymers, among other uses. On the other hand, the three main macromolecular components of lignocellulosic materials, cellulose, hemicelluloses and lignin, may be converted to fuels, solvents, drugs, platform chemicals and functional materials through a variety of processing technologies. Hence, by applying the biorefinery concept to efficient biomass conversion processes, one may envisage the development of products and processes that are able to compete with well-established conversion technologies such as those based on crude oil and coal, and this will pave the way for the implementation of a sustainable bioeconomy in our society, providing food, energy and environmental security for generations to come.

### Extraction of Lignocellulosic Materials Using Pressurized Fluid Technology

Supercritical fluids are an attractive alternative to the traditional use of organic solvents for extracting commercially relevant biomass constituents (Ibáñez et al., 2016). The main advantages of supercritical fluid extraction (SFE) relies on reducing or even eliminating the use of organic solvents, as well as reducing downstream purification processes mainly because the extraction solvent can be easily separated by simple expansion to atmospheric conditions (Reverchon and de Marco, 2006; Sahena et al., 2009). Furthermore, SFE may be fine-tuned to yield streams containing a high concentration of the desired compounds, therefore providing a much higher selectivity than conventional extraction methods.

As already mentioned, the most utilized supercritical fluid is CO_2_ due to its low cost, wide availability and safety characteristics (Brunner, 2005; Raveendran et al., 2005). Also, its solvent properties may be modulated by alterations in temperature and pressure conditions, as well as by adding modifiers or co-solvents for extracting a widely variety of chemicals from biomass. Additionally, many studies have been oriented to the use of pressurized hydrocarbon fluids such as propane and butane due to their high affinity for oils, waxes and greases (Correa et al., 2016; Fetzer et al., 2018; Gu et al., 2019).

The commercial demand for waxes and greases have increased considerably in recent years and this tendency is likely to increase. Conventional processes for the extraction of such lipophilic materials involve the use of large amounts of solvents whose main sources are petroleum (85%), organic synthesis (11%) and biomass (4%) (Attard et al., 2018). The main markets for waxes and greases are cosmetics and packaging, which are expected to value $ 9 billions by 2020, with Asia being the main supplier and the American and European markets the largest consumers.

Oil extraction from agricultural residues using compressed propane and scCO_2_ with and without co-solvents have already been studied by many with global yields normally comparable to those obtained using traditional solvent extraction procedures. For rice bran extraction, Sparks et al. (2006) reported 22 wt.% at the best extraction conditions using both propane (ambient temperature, 7.6 bar) and scCO_2_ (45°C, 350 bar). The addition of ethanol using scCO_2_ in a gas-expanded liquid scheme was evaluated by Juchen et al. (2019) for the extraction of oil from rice bran. Global yields close to 20 wt.% were obtained with CO_2_ consumption lower than 7 g⋅g^–1^ rice bran when using 2 g⋅g^–1^ of ethanol at 40°C and 100 bar.

Muangrat and Pongsirikul (2019) compared the scCO_2_ extraction of spent coffee grounds with traditional Soxhlet and an accelerated solvent extraction using propanol. scCO_2_ was carried out at 40–60°C and 175–225 bar for 1 and 3 h. No significant differences were found in oil yield for the two different extraction methods, with extraction yields residing in the range of 12 wt.%.

Carotenoids extraction of various vegetable waste matrices was investigated using scCO_2_ and ethanol as co-solvent (de Andrade Lima et al., 2019). The optimal process conditions were 59°C and 350 bar using 15.5% ethanol, scCO_2_ at a flowrate of 15 g⋅min^–1^ and a run time of 30 min. Fresh peels of sweet potato, tomatoes, apricot, pumpkin, peach and peppers were extracted with total carotenoids recoveries greater than 90% in all cases. The extraction of flavonoids from pomelo peels using scCO_2_ + ethanol presented good yields (over 2 wt.% at 80°C and 390 bar) and equivalent antioxidant activities compared to conventional Soxhlet extraction using ethanol (He et al., 2012).

The economic viability of supercritical extraction processes depends on factors such as the type of biomass to be mined, its presentation (particle size distribution), accessibility (pore volume and product location) and what products to obtain (Ibáñez et al., 2016). Therefore, supercritical extraction must be optimized to meet the pre-established process goals. Sometimes, process optimization is not so trivial because many different variables are involved. For this reason, experimental designs are applied to identify the optimal extraction conditions, therefore contributing to the economic viability of holistic biomass conversion technologies.

### Value Added Carbohydrates Derivatives

Several carbohydrate-based value-added products have been identified so far under the biorefinery concept, such as furfural, furfuryl alcohol, levulinic acid, ethyl levulinate, and butyl levulinate (Lomba et al., 2011). In general, many of these products are fermentation inhibitors, but they are also highly versatile chemicals that could help the viability of the entire production chain if their recovery and upgrading is maximized.

Furfural is a platform chemical with extensive industrial application that may be produced directly from pentose dehydration (Luo et al., 2019). Jaafari et al. (2019) investigated the kinetics of furfural production by acid-catalyzed flax straw liquefaction at temperatures between 200 and 325°C, pressures from normal to 60 bar and reaction times up to 120 min using γ-alumina, H-ZSM-5, and silica-alumina as catalysts. Among the conditions studied, the highest yield (66 wt.%) was obtained at 250°C and 60 bar using γ-alumina.

5-HMF is a platform chemical to produce renewable plastics and fuels. Bicker et al. (2003) studied the dehydration of fructose to 5-HMF in subcritical and supercritical water/acetone mixtures using sulfuric acid as catalyst at different concentrations (from 10 to 50 mmol⋅L^–1^). Variables such as temperature, pressure, time, and water/acetone ratios were assessed to improve reaction performance. High selectivities (about 75%) and almost theoretical conversions were obtained at lower water contents (10 vol.% in acetone), low temperatures (around 180°C) and high residence times (up to 120 s), while pressure was not influential. The use of sugarcane bagasse for the direct production of 5-HMF was studied by Iryani et al. (2013) under compressed water conditions (200–300°C for 3–30 min). The maximum 5-HMF production was obtained at 270°C for 10 min yielding 3.1 wt.%, while higher temperatures or reaction times led to 5-HMF decomposition or polymerization to formic acid and char, respectively. Although 5-HMF yields were relatively low, it must be emphasized that pretreatment was carried out with raw biomass under subcritical water conditions in the absence of an added catalyst. By contrast, Nguyen et al. (2016) obtained remarkably high 5-HMF yields by combining two sequential pretreatment steps involving dilute alkali extraction (3 wt.% NaOH at 60°C for 24 h) followed by oxidation with CrCl_3_⋅6H_2_O in the presence of 1-butyl-3-methyl imidazolium chloride, using a catalyst-to-solvent mass ratio of 1:25 and a biomass-to-solvent ratio of 1:20 at 120°C for 6 h. At the optimal condition, rice straw yielded 76 mol% while wood chips yielded 79 mol% in relation to the glucan content of the starting material biomass.

Levulinic acid is also considered a platform chemical with wide industrial use and it stands out as a connection between petrochemistry and biorefining applications (Bozell, 2010). This acid is not used just as a green solvent but also a precursor for fuels and additives. Lange et al. (2010) studied the production of valeric biofuels such as γ-valerolactone (GVL), ethyl levulinate (EL), methyl tetrahydrofuran (MTHF), and valeric acid (VA) using process conditions that resulted in high conversions and selectivity. As an example, these authors obtained a 95% conversion in LA by hydrogenation, with selectivity of the same magnitude for GVL when titanium oxide containing 1% platinum was used as catalyst in the presence of 40 bar of H_2_ and 200°C. Sequential reactions to obtain VA, EL, and MTHF were evaluated, products were obtained in high yields and properties of different process streams supported their use in several industrial applications. In another study, supercritical ethanol was applied for the autocatalytic conversion of levulinic acid to EL (Kothe et al., 2020). The best conversion of 80% was obtained using an ethanol-to-levulinic acid molar ratio of 9:1 at 280°C and 100 bar for 15 min.

### Economic Aspects of Supercritical Technologies Applied to Lignocellulose Materials

The feasibility of different supercritical processes in biomass utilization is evident, considering the environmental and technical benefits of supercritical fluids as green solvents for biomass conversion over classical pretreatment methods, as well as the advantages of direct fractionation of lignocellulose into fuels, products and materials without high consumption of chemicals nor costly separation steps. However, a thorough assessment of the economic viability of these technologies has not been well explored in the literature yet. Accordingly, some authors have compared the cost of producing saccharides through supercritical processes with classical pretreatment strategies such as dilute acid hydrolysis or organosolv fractionation.

Albarelli et al. (2016) studied the economic potential of various pretreatment processes for co-producing nanocellulose and ethanol in a sugarcane biorefinery. The results indicated that higher economic viabilities were achieved from processes that maximized nanocellulose yield, which was best for SO_2_-catalyzed steam explosion, followed by scCO_2_, organosolv, and organosolv + scCO_2_. However, this assessment did not include parameters such as utilities, energy and chemical costs as well as capital costs that are critical to identify the best profitability among the evaluated pretreatment processes.

Daza Serna et al. (2016) compared dilute acid hydrolysis with supercritical processes for rice husks using the Aspen Process Economic Analyzer V8.2 (Aspen Technology Inc., United States). A plant able to process 10 ton⋅h^–1^ of biomass was simulated until the enzymatic hydrolysis stage using data obtained experimentally and capital depreciation for 12 years of operation. Under these assumptions, the production cost was reduced from 1.88 USD⋅kg^–1^ using dilute acid pretreatment to 0.20 USD⋅kg^–1^ using scCO_2_ in the presence of water/ethanol as co-solvent. It was also hypothesized that, although costly is terms of energy demand, supercritical processes could facilitate the recovery of chemicals and energy by scCO_2_ expansion in a turbine to generate electricity. However, these hypothesis were not demonstrated in this simulation study.

Recently, Ortiz (2020) reported a general procedure for the techno-economic analysis of supercritical processes. This author emphasized that the accurate estimation of fluids transport and thermodynamic properties is critical for the economic assessment of such pretreatment technologies. Also, a critical analysis and a review of several techno-economic assessments applied for biofuel production was presented, mainly related to CO_2_, alcohol, and water at supercritical conditions. It was concluded that the estimated capital cost of such processes can be reduced by designing them together with mass and energy integration into a biorefinery concept. In any event, under the context of a biorefinery, it is important to remember that the economic viability of supercritical technologies relies on the maximum utilization of the lignocellulosic matrix, as much as on energy integration including co-generation strategies, given the high energy content of pressurized fluids.

## Concluding Remarks and Future Research Needs

The conversion of lignocellulosic materials to fuels and value-added chemicals offers an attractive solution for two modern problems: increased global energy demand and a rise in the environmental consciousness of modern society. However, lignocellulosic materials were designed by nature to withstand chemical or biological conversion, leading to the need of pretreatment technologies to increase its physical and/or chemical accessibility. Indeed, recalcitrance is the main drawback for the deployment of industrial-scale biomass conversion processes. In this regard, supercritical pretreatment techniques involving water, carbon dioxide, short chain alcohols or hydrocarbons, in the absence and presence of modifiers such as catalysts and/or co-solvents, are able to deliver high yields of useful products from lignocellulose without the release of hydrolysis and/or fermentation inhibitors. However, at severe temperature and pressure conditions, these same fermentation inhibitors may become high value-added platform chemicals and building blocks for other applications. Several types of biomass have been investigated so far using compressed fluid techniques and several of these studies have been optimized. As no set of pretreatment conditions seem to be universal, optimization studies are critical to improve reaction performance and process yields. The use of high temperatures and pressures is an economic bottleneck for the widespread utilization of supercritical pretreatment techniques in large scale, but the obtainment of stable, high value-added coproducts may justify its implementation, particularly when applied together with other technological routes that would contribute to the development of holistic approaches for the utilization of all possible valuable streams coming out of widely available agricultural, agro-industrial and forestry residues.

## Author Contributions

All authors contributed equally for the preparation of this manuscript.

## Conflict of Interest

The authors declare that the research was conducted in the absence of any commercial or financial relationships that could be construed as a potential conflict of interest.
